# Guyon tunnel syndrome secondary to excessive healing tissue in a child: a case report

**DOI:** 10.1186/1749-7221-3-16

**Published:** 2008-05-28

**Authors:** Aydıner Kalacı, Yunus Doğramacı, Teoman Toni Sevinç, Ahmet Nedim Yanat

**Affiliations:** 1 Dept. of Orthopaedics and Traumatology, Mustafa Kemal University, Faculty of Medicine, Antakya, Hatay, Turkey

## Abstract

We describe a case of an 8-year-old boy who developed a combined motor and sensory neuropathy of the distal ulnar nerve, after sustaining a superficial injury to the right flexor carpi ulnaris tendon at the level of the distal wrist crease. Guyon's canal syndrome is a very rare entity during childhood. We have noted only one prior description of this syndrome in the pediatric age group in a review of the English literature.

## Background

The distal ulnar tunnel, Guyon's canal, is 4–4.5 cm long. It begins at the proximal edge of the palmar carpal ligament and extends to the fibrous arch of the hypothenar muscles. The tunnel has frequently changing boundaries and does not have four distinct walls throughout its course. From proximal to distal, the roof consists of the palmar carpal ligament, the palmaris brevis, and the hypothenar fibrous and fatty tissue. The floor of the tunnel is made up of the flexor digitorium profundus, the transverse carpal ligament, the piso-hamate and piso-metacarpal ligament and the opponens digiti minimi. The flexor carpi ulnaris, the pisiform, and the abductor digiti minimi constitute the medial wall. The lateral wall is composed of the tendons of the extrinsic flexors, the transverse carpal ligament, and the hook of the hamate [1].

There are four levels in which the ulnar nerve may be compressed at the wrist and hand: 1) The main trunk of the nerve at the entrance to, or within Guyon's canal. These lesions produce sensory loss in the distribution of the superficial termination branch and weakness of all the ulnar-innervated intrinsic muscles. 2) The deep terminal motor branch of the ulnar nerve distal to Guyon's canal but proximal to the branches that innervate the abductor digiti minimi (hypothenar muscles). This produces weakness of all ulnar-innervated muscles of the hand without sensory loss. 3) The deep motor branch distal to the branches that innervate the abductor digiti minimi and the hypothenar muscles. This produces no sensory loss but there is weakness of all the ulnar innervated intrinsic hand muscles except the hypothenar muscles. 4) The superficial terminal sensory branch which produces sensory loss without muscle weakness [2].

Guyon's syndrome in the paediatric age group is extremely rare; a search of the literature in English yielded one case [3]. To our knowledge, this is the first reported case of an isolated Guyon's syndrome secondary to an injury of the flexor carpi ulnaris in a child.

## Case presentation

An 8-year-old boy presented to our clinic complaining of numbness of the little finger and the ulnar aspect of the ring finger. Ten days prior to presentation, the patient sustained a 1 cm laceration at the level of the distal wrist crease after falling on a piece of broken glass. On examination, he had weakness of abduction and adduction of the fingers. Movement of the thumb was unaffected.

The injury was managed at the emergency department by thorough wound irrigation. There was a partial irregular cut of about 30% of the radial aspect of the FCU with intact ulnar nerve and ulnar artery. The skin was sutured. After the primary management the patient was sent to our orthopaedic clinic for further follow up. The initial examination one week after the injury revealed a clean wound, no hematoma or swelling, normal sensation of the fifth and ulnar side of the fourth finger, and normal abduction and adduction of the digits. However a gradual numbness and weakness of intrinsic hand muscles was noted after 10 days that gradually worsened. On subsequent follow up a total ulnar nerve deficit was noted distal to the injury, at the wrist level involving motor and sensory branches.

Three weeks after the initial injury he developed marked weakness of all ulnar supplied intrinsic muscles with total sensory loss at the fifth and the ulnar side of the fourth fingers. Due to the progressive nature of his symptoms, exploration and decompression of the Guyon's canal was done under general anaesthesia. Exploration revealed normal healing of skin and subcutaneous tissue with excessive scar tissue at the radial edge of the FCU which spanned the ulnar nerve, narrowing the entrance of Guyon's canal and causing severe compression and cicriatrical constriction of the nerve.

**Figure 1:**
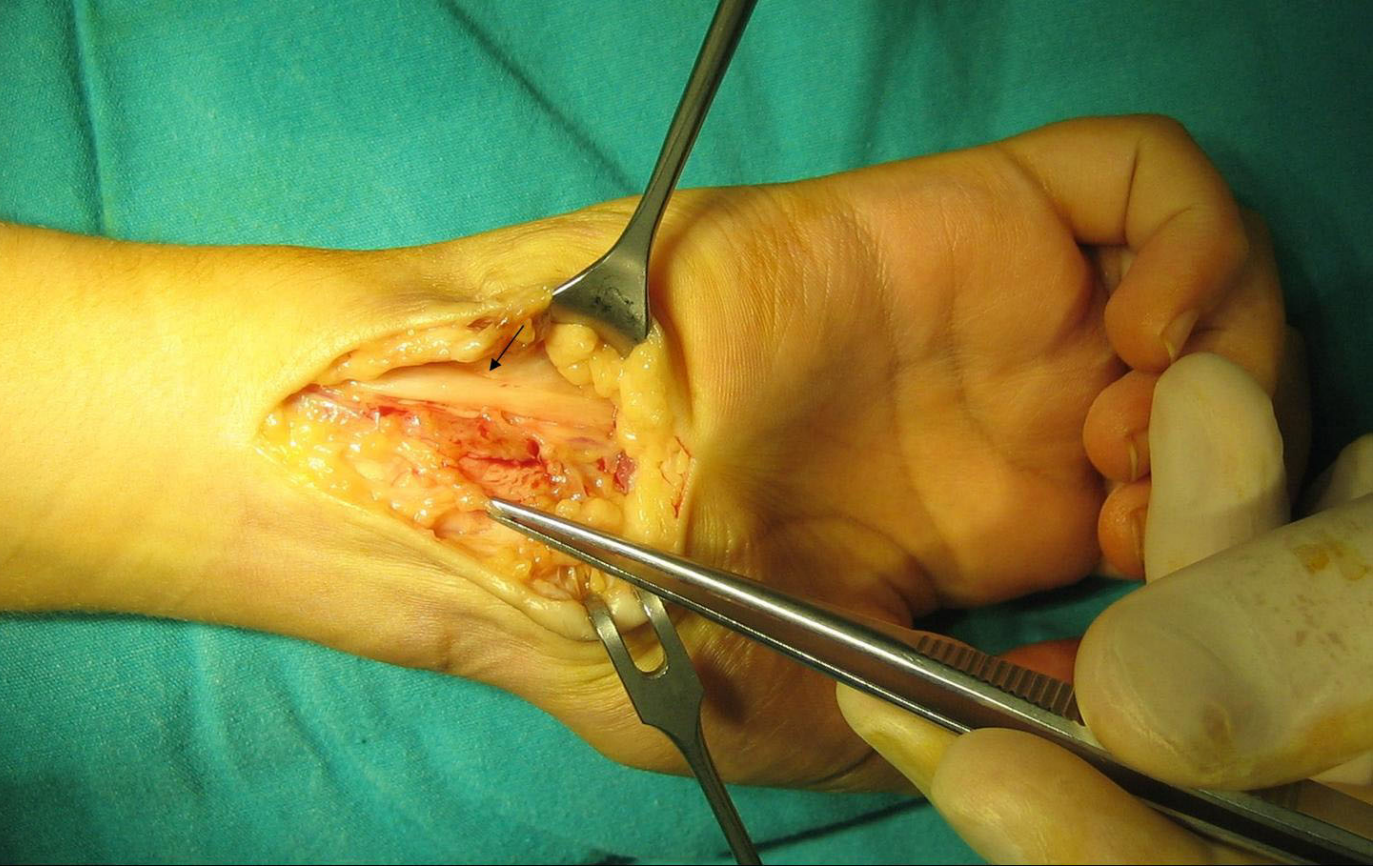
The ulnar nerve after release of the adhesion at the entry of the Guyon's canal; there is marked compression and some proximal bulging of the nerve at the site of the adhesion (Black arrow).

The ulnar nerve was completely intact (Fig. 1). No organized hematoma or lesion of ulnar artery was observed. Adhesions were released, excised and Guyon's canal was completely released. Physiotherapy was started immediately post-operatively, encouraging the patient to move the wrist and fingers. Sensation was markedly improved by the first post-operative day with nearly complete return of motor function at one week. At three months, the recovery was complete.

## Discussion

Intrinsic lesions (ganglia, lipoma, cysts, anomalies of ligaments or muscles, ulnar artery aneurysms, fracture of hook of the hamate) as well as extrinsic pathologies (chronic repetitive trauma) can damage the terminal superficial and/or deep branches of the ulnar nerve at the wrist and at the hand leading to distinct clinical features [2-4].

The most common lesion, to the proximal Guyon's canal (Type 1), is characterised by sensory loss at the ulnar portion of the hand and weakness of all ulnar intrinsic hand muscles (mixed sensory-motor dysfunction), whereas a more distal lesion within Guyon's canal (Types 2 and 3) causes an isolated palsy of the deep terminal motor branch without sensory loss (pure motor dysfunction) [2,4,5]. Numerous occupations and pastimes that are associated with ulnar neuropathy have been described in the literature. These include bicycle riding, pizza cutting, and prolonged playing of video games, karate, and intensive use of a computer mouse [6,7]. Traumatic causes of ulnar neuropathies at the wrist include fractures of the distal radius or ulna and of the carpal bones [8]. In our case, ulnar nerve compression was secondary to excessive cicatricial tissue from a partial laceration of the flexor carpi ulnaris tendon at the proximal margin of Guyon's canal, which was not repaired.

To our knowledge this is the first case in the literature published in English language which reports Guyon's syndrome secondary to excessive healing tissue following partial tendon injury in the paediatric age group. This may be attributable to the high healing potential in paediatric patients. In addition, the experience of this case presents a challenge to the current dogma of withholding repair of tendon lacerations of less than 60% of the tendon's cross-sectional area [9].

## Conclusion

In the paediatric age group, glass penetrating injuries in proximity to neurovascular structures are best explored irrespective of distal neurologic deficits. Clearly the surgical exploration should not supplant a thorough preoperative clinical examination. The potential for otherwise missing incomplete tendon and vascular injuries is high. It also gives one the opportunity to more thoroughly irrigate the wound and evaluate hematomas.

A neuroma-in-continuity found at delayed exploration is more difficult to treat than the original acute injury. Surgical exploration is indicated in all lacerations of the hand and upper extremity unless the level of injury is sufficiently superficial to enable exlusion of damage to vital structures in the emergency department. The experience of this case presents a challenge to the current dogma of indications for tendon repair, especially in the paediatric population.

## Competing interests

The authors declare that they have no competing interests.

## Authors' contributions

AK carried out the operation, YD participated in the sequence alignment and drafted the manuscript, TTS and ANY participated in the design and coordination of the manuscript. All authors read and approved the final manuscript.

## Consent

Written informed consent was obtained from the patient for publication of this Case report and accompanying images. A copy of the written consent is available for review by the editor-in-chief of this journal.
